# FEASIBILITY AND VALIDITY OF WEARABLE TECHNOLOGY FOR ASSESSING MOBILITY AMONG HOSPITALIZED OLDER ADULTS

**DOI:** 10.1093/geroni/igad104.2814

**Published:** 2023-12-21

**Authors:** Paulo Roberto Carvalho do Nascimento, Renata Kirkwood, Mylinh Duong, Lauren Griffith, Marla Beauchamp

**Affiliations:** McMaster University, Hamilton, Ontario, Canada; McMaster University, Hamilton, Ontario, Canada; McMaster University, Hamilton, Ontario, Canada; McMaster University, Hamilton, Ontario, Canada; McMaster University, Hamilton, Ontario, Canada

## Abstract

The need for strategies to promote mobility during acute care hospital stays among older adults is increasingly recognized. Wearable technologies show great potential for this purpose. The objective of this study was to determine the most accurate wearable sensor device and location for capturing in-hospital mobility in older patients. Twenty-five older adults (79.6±8.1 years) admitted to a hospital medical care unit volunteered to test the feasibility and concurrent validity of the ActiGraph wGT3X-BT, Mox1, MetaMotionC and Fitbit Versa. All 25 enrolled patients wore the devices simultaneously on the wrist, hip, and ankle while undertaking a mobility activity protocol consisting of supine lying, sitting, and standing tasks. Participants also performed the Timed Up and Go (TUG) and 10-meter walk test (10MWT) wearing the devices. A trained physiotherapist supervised the performance of the tasks. Most participants (58%) preferred to wear the device on the ankle. Overall, the ActiGraph and Mox1 were the easiest to set up and download. The ActiGraph was the most reliable device, retrieving 100% of the collected data. Regarding body posture, the thigh-worn ActiGraph algorithm accurately classified 78% of sitting and lying postures, as well as 84% of standing postures, indicating high overall accuracy. After implementing the recommended low-frequency filter extension for slower gait speeds, the ankle-worn ActiGraph showed the narrowest limits of agreement and observations closer to zero for step count during the TUG and the 10MWT. These findings lay the foundation for subsequent work to validate wearable devices for monitoring “free-living” mobility in this population.

